# Whole body MR-PET: a new internal dosimetry method for radiation transport calculation from biokinetic model data

**DOI:** 10.1186/2197-7364-1-S1-A78

**Published:** 2014-07-29

**Authors:** Ana Nunes, Francisco Alves, Miguel Patrício

**Affiliations:** Institute of Nuclear Sciences Applied to Health (ICNAS), University of Coimbra, Kragujevac, Portugal; College of Health Technology - Polytechnic Institute of Coimbra, Kragujevac, Portugal; Laboratory of Biostatistics and Medical Informatics, IBILI – Faculty of Medicine, University of Coimbra, Kragujevac, Portugal

In order to ensure the safe usage of new radiopharmaceuticals in Positron Emission Tomography (PET), it is necessary to quantify the doses delivered to the organs and tissues within the patients’ bodies. A framework that allows estimating the dose delivered by PET has been established by the MIRD Committee [1, 2] and ICRP [3]. Although this covers the most important terms and concepts in Internal Radiation Dosimetry (IRD), it does not provide a detailed guide to assist in the development of a full dosimetric study. We discuss the development, implementation, assessment and validation of an accurate method for IRD studies of PET radiotracers.

We undertook a systematic review [4, 5] of previous PET dosimetry studies to identify the current biodistribution data acquisition and analysis methodologies. The methods reported were compared, particularly in terms of the collection of biokinetic data and the estimation of dose results. As a wide heterogeneity of approaches was found, it is important to assessthe impact of different choices in the internal dose calculation protocol. In particular, we quantify the variation in internal dose with different assumptions regarding organ activity uptake - namely, instantaneous uptake *vs*. different methods to extrapolate to zero activity at time zero. For that purpose, whole-body ^11^C-Raclopride PET data have been acquired and the corresponding internal doses estimated.
